# Genetic variations and clinical implications of B-thalassemia in Iraqi population

**DOI:** 10.1371/journal.pone.0344034

**Published:** 2026-03-04

**Authors:** Ayman Ziadoon Jawad, Meryam Chelly, Salah Hashim AL-Zuhairy, Hanen Bouaziz

**Affiliations:** 1 Department of Medical Laboratory Technologies, Al-Manara College for Medical Sciences, Maysan, Iraq; 2 Department of Engineering, University of Messina, Messina, Italy; 3 Laboratory of Environmental Toxicology-Microbiology and Health, Faculty of Sciences, University of Sfax, Sfax, Tunisia; 4 Department of Pediatrics, College of Medicine, Mustansiyriah University, Baghdad, Iraq; Shaheed Rajaei Cardiovascular Medical and Research Center: Rajaie Cardiovascular Medical and Research Center, IRAN, ISLAMIC REPUBLIC OF

## Abstract

β-thalassemia is a prevalent genetic disorder in Iraq, leading to significant health issues due to reduced hemoglobin production. The DNA sequencing technique was used to explore genetic variations and their clinical implications. Our findings have the potential to inform diagnosis, guide targeted therapeutic approaches, and enhance genetic counseling to reduce long-term morbidity in affected individuals. Peripheral blood samples were collected from 100 patients for analysis. Quantitative measurements included complete blood count (CBC), ferritin, parathyroid hormone (PTH), lactate dehydrogenase (LDH), 25‑hydroxyvitamin D, phosphorus, calcium, and bone mineral density (BMD) measured by dual-energy X-ray absorptiometry (DXA). We detected 18 β-globin mutations in β-thalassemia patients by direct Sanger sequencing, of which two were novel (HBB:c.315 + l08A>G and HBB:c.316-151A > G). Additionally, four mutations (IVS-II-1G > A, IVS-II-5G > C, IVS-I-110G > A, and HBB:c.440A > C) have been previously reported as pathogenic. This research pinpointed four β-globin gene pathogenic mutations as having significant associations with clinical parameters. Hematological parameters (HGB, WBC, RBC indices, RBC count) and biochemical/metabolic markers (phosphorus, PTH, LDH, ferritin, vitamin D3, calcium, ALP) exhibited strong statistical differences (*p* < 0.001–0.020) across mutation groups. Bone health markers (BMC, BMD) and red blood cell indices (MCH, MCHC, MCV, MPV) also showed significant variation (*p* < 0.001–0.002). In contrast, platelet count (PLT) did not differ significantly (*p* = 0.331). These findings highlight mutation specific impacts on hematological, metabolic, and skeletal systems in the studied population.

## Introduction

β-thalassemia is a hereditary hematological disorder resulting from mutations in the HBB gene, which reduces or eliminates production of the globin protein chain for hemoglobin. This disrupted synthesis of β-globin chains causes an imbalance between α and β globin chains, which leads to ineffective erythropoiesis and hemolytic anemia. This condition leads to clinical symptoms that range from mild β-thalassemia minor to severe anemia requiring transfusions in cases of β-thalassemia major [1].

It is important to mention that the number of pathogenic mutations of the HBB gene has exceeded 400 worldwide, and they can be point mutations and small insertions or deletions, as well as relatively common structural rearrangements, which contribute to variability in disease severity and responses to treatments [2].

Knowledge of the particular sequence of the HBB gene is important for several reasons. The first reason is a proper diagnosis and genetic counseling for the affected families. Second, it may guide the understanding of the pathophysiology of the disease and, therefore, possible treatments. Third, characterization of mutations may be useful in designing new forms of treatment, such as gene therapy and new pharmacological agents to induce fetal hemoglobin [3]. The current study aimed to analyze the spectrum of the HBB gene mutations in patients with β-thalassemia. We hope to identify these mutations by using the Sanger sequencing method and to relate them to clinical phenotypes. The results of this study are expected to provide new insight and a better understanding of the pathology of β-thalassemia, leading to improvements in treatment and the introduction of new therapies. Through this study, it is also our aim to bring into the light the genetic heterogeneity of β-thalassemia, highlighting the necessity of an individualized approach in diagnostic and personalized therapeutic strategies guided by the patient’s specific genetic background [4].

HBB mutations exist at different frequencies in the Iraqi population. The ⅣS-I-lG > T mutation, which is common in beta-thalassemia, and the HbS mutation (Glu6Val) that is characteristic of sickle cell anemia. Furthermore, some other mutations, like the ⅣS-II-1 G > A have been reported and could be unique or more frequent in other Iraqi subpopulations.

## Methodology

### Sampling

The study included 100 children and adolescents aged 5–16 with β-thalassemia, who were treated at the Women’s & Children’s Hospital in Maysan and Al-Karama Teaching Hospital in Baghdad, Iraq, as well as 100 healthy participants matched by both gender and years of age. Samples of whole blood were obtained from 17 March 2023 to 4 February 2024. The research protocol was approved by Al-Manara University’s ethics board (Department of Medical Laboratory Techniques, approval E/580, dated December 28, 2022). All participants or their parents or legal guardians signed consent forms before joining the study.

### Genomic DNA extraction, PCR and sequencing

Genomic DNA of peripheral blood samples was extracted using the ReliaPrep Blood gDNA Miniprep system (Cat. No. A5081, Promega Corp., USA) according to the manufacturer’s instructions. The DNA was then analyzed with a NanoDrop 8000 (Thermo Scientific, USA) to confirm that it was of a concentration suitable for further use.

The amplification of the β-globin gene was performed with two different PCR mixes: PCR mix I with equimolar concentrations of the forward primer 5′-ACTGATGGTATGGGGCCAAG**-**3**′** and reverse primer 5′- AATAGTAATGTACTAGGCAGACTGT- 3′. PCR mix II comprised forward primer: 5′- GTATCATGCCTCTTTGCACC- 3′ and reverse primer: 5′-GGAGAAACCATCTCGCCGTAA-3′, that were virtually designed by the NCBI-primerBLAST web tool [5,6].

The 50 μL PCR reaction mixture contained 25 μL of GoTaq Long PCR master mix (Cat. No. M4021, Promega Corp., USA) and 2 μL of 10 μM PCR primer mix (I or II). The PCR conditions included an initial denaturation at 94°C for 5 minutes followed by 38 cycles of 94°C for 30 sec, 60°C for 30 sec, and 72°C for 1 min. The reaction was finished by a final at 72°C for 5 min. Amplified products were then separated on agarose gel by gel imaging. The gel presented distinct bands of 1,056-bp product for primer mix I and 936-bp product for mix II. The two PCR products were purified and sequenced with the BigDye terminator (V.3.1) and the right primers. DNA sequencing was outsourced to Macrogen Inc. (South Korea).

### Hematological and biochemical parameters

The red blood cell (RBC) indices were measured on an XN-330 hematological analyzer (Sysmex, Japan). Serum ferritin and parathyroid hormone (PTH) levels were detected by Mini Vidas (Biomerieux, France). A quantitative sandwich ELISA was utilized to measure the levels of lactate dehydrogenase (LDH) and 25-hydroxy vitamin D using an ELx800 absorbance microplate reader. Phosphorus and calcium quantities in serum were detected using a spectrophotometer (APEL, Japan) by the ammonium molybdate endpoint method. Bone mineral density (BMD) was measured at the lumbar spine (L1–L4) and femoral neck using dual-energy X-ray absorptiometry (DXA) for patients aged 10 years and older. BMD is reported in g/cm^2^, with corresponding pediatric reference values expressed as Z-scores based on age- and sex-specific norms. Mean (SD) BMD and Z-scores were compared between patients and controls.

### Statistical analysis

The results were statistically analyzed using SPSS version 23. Comparisons and differences were tested by the x^2^ test. A t-test and analysis of variance were used for identification of statistical differences between the ELISA results, as well as at a P-value ≤0.05.

## Results

In this study, 100 patients participated 53 males and 47 females. The frequency of β-thalassemia types showed a significant difference (*P* = 0.002), with 67% of individuals diagnosed with β˗thalassemia major and 33% with the intermediate type. The comparative analysis of hematological parameters of both case and control groups revealed significant differences in multiple biomarkers. As shown in S1 Table in S1 File, the effect of β-thalassemia on hematological parameters.

The biochemical analysis (S2 Table in S1 File) revealed significant alterations in thalassemia patients (Case) compared to healthy controls. Ferritin levels were markedly elevated in patients (*p* = 0.001), consistent with iron overload, while alkaline phosphatase enzyme (ALP) was significantly higher (*p* ₌ 0.001), suggesting hepatic or bone involvement. Calcium levels showed a modest but significant increase (*p* = 0.020), with both gender (*p* = 0.03) and age (*p* = 0.044) contributing to variability. Notably, lactate dehydrogenase (LDH) was unexpectedly lower in patients (*p* = 0.001), diverging from typical hemolytic profiles and warranting further investigation into potential metabolic adaptations. Parathyroid hormone (PTH) levels were reduced (*p* = 0.001), accompanied by elevated phosphorus (*p* = 0.001) and pronounced vitamin D3 deficiency (*p* = 0.001), collectively indicating disrupted bone-mineral homeostasis.

The study found that, although height and BMI were similar between thalassemia patients and healthy controls, patients had significantly lower bone mineral content and density, with these deficits increasing with age. Z-scores were also reduced in patients and affected by age. No notable differences were observed in weight or body surface area, as shown in S3 Table in S1 File.

The analysis of β-globin gene sequence using a reference sequence of the β-globin gene (NC_000011.10) by UniPro UGENE v52.0 found the presence of 168 mutations of 18 different types in 100 patients with β-thalassemia: HBB:c.˗113A > G, HBB:c.9T > C, HBB:c.˗113A > G, HBB:c.41C > T, IVSII˗5G > C, S-I-110G > A, HBB:c.189T > C, IVS-II˗lG > A, IVS-II˗16G > C, IVS-II˗74T > G, HBB:c.315 + l08A>G, IVS-II-666C > T, HBB:c.316-151A > G, HBB:c.316-143A > G, HBB:c.316-31C > T, HBB:c.440A > C, 3’UTR*4C > T, 3’UTR + 101G > C and HBB:c.*3l6A>C, as shown in Table 1. The mutation profiles were evaluated with the HbVar [7], Varsome [8], and ClinVar (NCBI) [9] databases. This study adhered to the sequence variant standards established by the Human Genome Variation Society (HGVS) and complied with IUPAC naming conventions [10]. These mutations are available in ClinVar-NCBI (S4 Table in S1 File), which lists the accession numbers (SCV006104260–SCV006104277) and ClinVar submission data.

**Table 1:** The list of the identified mutations in the HBB gene of the study.

Variation ID	dbSNP rs	HGVS-c	HGVS-g	Location	Classification	No. (%)
HBB: c.-113A > G	rs1354742084	NR	NC_000011.10:g.5227134T > C	promoter	VUS	1 (1.00)
HBB:c.9T > C	rs713040	NM_000518.5:c.9T > C	NC_000011.10:g.5227013A > G	exon 1	Benign	1 (1.00)
HBB: c.41C > T	rs35203747	NM_000518.5:c.41C > T	NC_000011.10:g.5226981G > A	exon 1	VUS	8 (8.00)
1VS-1˗5G > C	rs33915217	NM_000518.5:c.92 + 5G > C	NC_000011.10:g.5226925C > G	intron 1	Pathogenic	30 (30.00)
IVS I-110G > A	rs35004220	NM_000518.5:c.93-21G > A	NC_000011.10:g.5226820C > T	intron 1	Pathogenic	22 (22.00)
HBB:c.189T > C	rs770878031	NM_000518.5:c.189T > C	NC_000011.10:g.5226703A > G	exon 2	Benign	3 (3.00)
IVS II-1G > A	rs33945777	NM_000518.5:c.315 + 1G > A	NC_000011.10:g.5226576C > T	intron 2	Pathogenic	32 (32.00)
IVS-II-16G < C	rs10768683	NM_000518.5:c.315 + 16G > C	NC_000011.10:g.5226561C > G	intron 2	Benign/likely Benign	10 (10.00)
IVS II-74T > G	rs7480526	NM_000518.5:c.315 + 74T > G	NC_000011.10:g.5226503A > C	intron 2	Benign	5 (5.00)
HBB:c.315 + l08A>G	novel SNP	NM_000518.5:c.315 + 108A > G	NC_000011.10:g.5226469T > C	intron 2	Likely Benign	4 (4.00)
IVS-II-666 C > T	rs1609812	NM_000518.5:c.316-185C > T	NC_000011.10:g.5225911G > A	intron 2	Benign	22 (22.00)
HBB:c.316-151A > G	novel SNP	NM_000518.5:c.316-151A > G	NC_000011.10:g.5225877T > C	intron 2	Likely Benign	2 (2.00)
HBB:c.316-143A > G	rs1589891738	NM_000518.5:c.316-143A > G	NC_000011.10:g.5225869T > C	intron 2	Likely Benign	7 (7.00)
HBB:c.316-31C > T	rs578102677	NM_000518.5:c.316-31C > T	NC_000011.10:g.5225757G > A	intron 2	Likely Benign	2 (2.00)
HBB:c.440A > C	rs33954264	NM_000518.5:c.440A > C	NC_000011.10:g.5225602T > G	exon 3	Pathogenic	1 (1.00)
HBB:c.*4C > T	rs372503204	NM_000518.5:c.*4C > T	NC_000011.10:g.5225594G > A	3’UTR	VUS	11 (11.00)
3’ UTR + 101 G > C	rs12788013	NM_000518.5:c. *233G > C	NC_000011.10:g.5225365C > G	3’UTR	Benign	5 (5.00)
HBB:c.*316A > C	rs7110263	NM_000518.4:c.*316A > C	NC_000011.10:g.5225282T > G	3’UTR	Benign	2 (2.00)

VUS = Uncertain Significance, UTR = Untranslated region, NR = not reported.

The results indicated that only four out of the eighteen types of mutations, specifically IVS-I-5G > C, IVS-I-110G > A, IVS-II-666C > T and 3’UTR + 101G > C were identified in patients with β-thalassemia in Iraq from prior studies.

Confirmatory genetic analysis revealed homozygous pathogenic variants in the HBB gene (IVS-I˗5 G > C; IVS-I˗110G > A; IVS-II˗lG > A; HBB:c.440A > C), located at introns 1 and 2 and exon 3, consistent with a biallelic inheritance pattern.

This study identified two novel deep intronic variants in the HBB gene, HBB:c.315 + 108A > G and HBB:c.316-151A > G. Both variants reside in non-canonical splice regions distal to essential splice motifs, raising the possibility of aberrant splicing. Specifically, HBB:c.315 + 108A > G is positioned 108 nucleotides downstream of the end of exon 2 (within intron 2), whereas HBB:c.316-151A > G lies approximately 151 nucleotides upstream of exon 3 (also within intron 2). In silico pathogenicity assessments using CADD (GRCh38-v1.7) yielded low scores for HBB:c.315 + 108A > G and HBB:c.316-151A > G, 0.098 and 0.140, respectively, both well below pathogenic thresholds (≥20). PhyloP conservation analyses similarly indicated low evolutionary constraint at these loci, with scores of −4.32 for HBB:c.315 + 108A > G and −3.115 for HBB:c.316-151A > G, consistent with non-conserved nucleotide positions. Clinical and predictive data from ClinVar (accessions SCV006104261 and SCV006104263) and MutationTaster support a benign classification for both alleles. Although deep intronic variants can, in theory, disrupt splicing via cryptic site activation, there is no empirical or functional evidence to support such a mechanism for these variants; their locations outside key splicing regulatory elements, combined with the lack of pathogenic signals across multiple predictive frameworks, argue strongly against a role in β-thalassemia pathogenesis.

Interpretation according to ACMG/AMP criteria of HBB:c.315 + 108A > G meets criteria to be classified as likely benign for beta-thalassemia (BP4_strong, PM2_supporting); HBB:c.316-151A > G meets criteria to be classified as likely benign for beta-thalassemia (BP4_strong, PM2_supporting) [11]. Overall, the integrated genomic, evolutionary, and in silico evidence indicates that both HBB:c.315 + 108A > G and HBB:c.316-151A > G are unlikely to contribute to β-thalassemia pathogenesis; further functional studies would be required to definitively exclude any cryptic splicing effects, but current data favor a benign interpretation.

**Fig 1:**
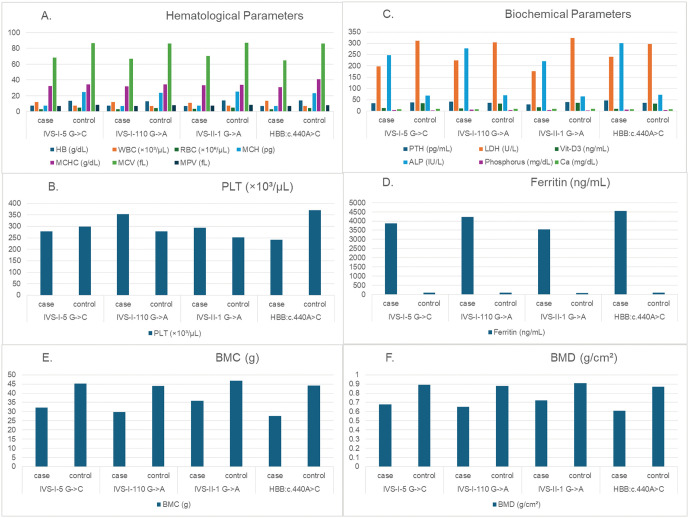
Associations between β-globin mutations IVS-I-5G > C, IVS-I-110G > A, IVS-II-lG > A, HBB:c.440 A > C and clinical parameters A, B, C, D, E and F figure sections. Significant variations were observed across mutation groups for hemoglobin (HGB), white blood cell count (WBC), phosphorus, parathyroid hormone (PTH), lactate dehydrogenase (LDH), ferritin, vitamin D3, calcium (Ca), alkaline phosphatase (ALP), red blood cell indices (MCH, MCHC, MCV, MPV), red blood cell count (RBC), bone miniral content (BMC), and bone mineral density (BMD). Platelet count (PLT) showed no significant differences (p = 0.331) among groups.

This study reported that 119 the HBB mutations (70.83%) were homozygous, while 49 mutations (29.18%) were heterozygous. The results analysis revealed significant associations between four mutations (IVS-I-5G > C, IVS-I-110G > A, IVS-II-lG > A, and HBB:c.440A > C and multiple clinical parameters (Fig 1). Hemoglobin (HGB, p < 0.001), white blood cell count (WBC, p = 0.007), phosphorus (p < 0.001), parathyroid hormone (PTH, p < 0.001.), lactate dehydrogenase (LDH, p < 0.001), ferritin (p. < 0.001.), vitamin D3 (*p.* < 0.001), calcium (Ca, p = 0.020), alkaline phosphatase (ALP, p < 0.001), red blood cell indices (MCH, MCHC, MCV, MPV, all p < 0.001), RBC count (p < 0.001), bone mineral content (BMC, p = 0.002), and bone mineral density (BMD, p < 0.001) showed statistically significant variations across mutation groups. In contrast, platelet count (PLT, p = 0.331) exhibited no significant differences among the groups.

## Discussion

The biochemical profile described for the thalassemia patients provides valuable insights into the complex metabolic and physiological alterations associated with the condition. The biochemical profile described for the thalassemia patients provides valuable insights into the complex metabolic and physiological alterations associated with the condition. Elevated alkaline phosphatase (ALP) levels in these patients highlight concurrent hepatic and skeletal involvement. Hepatic iron deposition contributes to fibrosis and cirrhosis [12], while bone marrow expansion in thalassemia stimulates osteoclast activity, increasing bone-specific ALP release**.** These dual mechanisms underscore the systemic nature of iron-mediated pathology. A paradoxical finding in some patients is the coexistence of reduced parathyroid hormone (PTH) levels and elevated calcium. Iron toxicity may impair parathyroid gland function, suppressing PTH secretion [13]. Concurrently, heightened bone resorption driven by cytokines like RANKL in response to ineffective erythropoiesis [14] and disrupted vitamin D metabolism due to hepatic or renal iron overload may explain the unexpected hypercalcemia [15,16]. Notably, lactate dehydrogenase (LDH) levels often deviate from the typical hemolytic profile, appearing paradoxically low. This phenomenon may arise from intramedullary destruction of erythroblasts (releasing less LDH than intravascular hemolysis) [17], chronic hypoxia-induced metabolic reprogramming [18], or oxidative inactivation of LDH isoenzymes [19]. The findings of this study improve understanding of β-thalassemia, especially in the Iraqi population. It is characterized by reduced or absent production of β-globin chains resulting in hypochromic, microcytic anemia of variable severity. Given the prevalence of this genetic disorder in Iraq, the detection 0f novel mutations in the HBB gene, specifically the HBB:c.315 + 108A > G and HBB:c.316-151A > G variants, emphasizes the genetic diversity and the need for tailored diagnostic and therapeutic approaches. Our study successfully identified 18 distinct mutations within the HBB gene in the sampled population of 100 patients. With only a few of these mutations previously documented in Iraqi patients, the introduction of novel candidates broadens the genetic landscape of β-thalassemia in this region and potentially reveals unique population-specific variants. This result highlights the relevance of ongoing genetic investigation and mutation specification because this may contribute to an increased value in the diagnostic accuracy and the personalization of treatment strategies.

The current study describes five pathogenic thalassemia mutations: IVS-II-lG > A, IVS-I*-*5G > C, IVS-I-110G > A, and HBB:c.440A > C; with IVS-II-lG > A mutation, was detected in patients residing in Northeast of Iraq (Sulaymaniyah), of whom 52% were homozygous for this mutation [20]. This variant is common in Iraq 52.04% [21] and in nearby areas; its frequencies are 20% in Jordan, 15% in Saudi Arabia, 4% in Syria, and 29% in Kuwait, which is higher than the other countries [22]. As well as it was characterized as the most frequent mutation in Iranian β-thalassmia patients, with the frequency rate of 61% from 734 total cases researched [23].

Also, in the current study, mutation IVS-I-5G > C was found in 30% of patients. This polymorphism has also been reported in some areas in Iraq with different rates: Al Muthanna 53.8% [21], Baghdad 26.3%, Babylon 10%, Basrah province 21.7% [24]. Variation in prevalence of this mutation in different regions of Iraq might have been due to potential introduction of this mutation from the neighboring countries with prevalence of IVS-I-5G > C; Iran (66.2%) [25], Saudi Arabia (19%) [26], Kuwait (17%) [27], and Turkey (2.1%) [28] known to have high rates of ⅣS-I-5G > C mutation. Remarkably, the IVS-I-5G > C mutation is the most common among β-thalassemia cases in Bangladesh (81.5%) [29].

The IVS-I-110G > A (HBB:c.93-2lG > A) mutation is a well-documented pathogenic variant associated with β-thalassemia, particularly in populations across the Mediterranean, Middle East, and neighboring regions of Iraq. Its prevalence varies significantly across countries, reflecting historical migration patterns, founder effects. In this study, IVS-I-110G > A was record at a frequency of 19% (19 patients). The patients with IVS-I-110(G > A) mutation showed a broad variety in clinical symptoms ranging from mild to severe. The incidence of the IVS-I-110G > A mutation (β ⁺ -thalassemia) exhibits varying prevalence across Iraq’s neighboring countries, with the highest frequency observed in Turkey (30–40% of β thalassemia alleles) [28], followed by Iran (15–20%) [25,30], Syria (5–10%) [22], Saudi Arabia (5–10%) [31], and Jordan (3–8%) [22].

This study reports one case causes β ⁺ -thalassemia (mild-moderate anemia to intermedia). The HBB:c.440A > C mutation (historically termed codon 142 (TAC > TCC) is a rare β ⁺ -thalassemia allele globally and is not commonly reported as a predominant mutation in Middle Eastern populations, including Iraq’s neighbors [22,32].

The HBB:c.440A > C mutation detected in the present study in Iraq is a point mutation in the HBB gene. This mutation changes the second nucleotide of codon 147(CAC > CCC), affecting the amino acid change of histidine to proline at the 147 (p.His147 Pro) position in the C-terminus of the beta-globin chain [33]. The C-terminus is important for stabilizing Hb and for facilitating association with alpha-globins to form a functional tetramer [34]. The substitution of histidine, a polar residue, with proline, a rigid, cyclic amino acid known to disrupt alpha helical structures, likely compromises the structural integrity of the beta-globin chain [35]. Such destabilization may impair hemoglobin tetramer formation or promote degradation of unstable hemoglobin, potentially leading to a hemoglobinopathy or a thalassemia-like phenotype [36]. The HBB:c.440A > C variant is classified as a rare missense mutation, with limited reported cases globally [37]. Novel mutations like HBB:c.440A > C may contribute to the regional burden of hemoglobin disorders; particularly in populations with elevated rates of consanguinity, autosomal recessive conditions are more commonly observed [38]. Targeted carrier screening programs are essential to recognize individuals at increased risk, to enable informed reproductive decisions, and to decrease disease burden through genetic counseling.

The correlation of individual mutations with different clinical variables is an essential aspect to be addressed. The significance of the hematological indices, biochemical and bone health parameters between mutations also underscores the mutation-dependent effects on patient health [39]. The significant difference in hemoglobin, WBC, ferritin, and PTH levels among mutation groups indicates the possibility that some mutations could aggravate the symptoms or even affect the severity of the disease phenotype [40,41].

In addition, the strong associations between certain mutations and BMD and BMC reveal an additional aspect of β-thalassemia care, where these patients commonly acquire metabolic bone disease because of iron overload and hormone dysregulation. These results underline the importance of early detection of metabolic and skeletal complications in the overall management of β-thalassemia [42].

Our research also provides a foundation for future investigations into potential therapeutic interventions. By identifying specific mutations linked to disease severity, it may be possible to develop more precise therapies, including gene editing techniques such as CRISPR/Cas9, or pharmacological agents aimed at increasing fetal hemoglobin levels. Moreover, the advent of gene therapy as a treatment option for β-thalassemia presents new opportunities for patients, particularly those with severe forms of the disease.

## Conclusion

The present study supports association findings of mutation types in the HBB gene with the clinical phenotype of β-thalassemia in the Iraqi patients. The detection of novel mutations both contributes to the genetic repository of the disease and also emphasizes the importance of localized diagnostic and treatment procedures. We propose the development of integrated genetic counseling programs, clinical care pathways and additional research around long-term care for patients with this multi-faceted disorder so as to improve the care of patients and quality of life. Additional investigations are required to elucidate the molecular mechanisms implicated in these correlations and to confirm this relationship in larger populations.

## Supporting information

S1 File **S1 Table.** Hematological parameters in patients versus controls. **S2 Table.** Biochemical parameters in patients versus controls. **S3 Table**. Anthropometric characteristics and bone mineral parameters in t β-halassemia patients versus healthy controls. **S4 Table**. Identified HBB gene mutations in the study (n = 100) with ClinVar data accession (SCV006104260-SCV006104277), clinical significance, frequency, and minimal data associated with ClinVar submission.
(RAR)
